# Atypical Ubiquitination and Parkinson’s Disease

**DOI:** 10.3390/ijms23073705

**Published:** 2022-03-28

**Authors:** Olga Buneeva, Alexei Medvedev

**Affiliations:** Institute of Biomedical Chemistry, 10 Pogodinskaya Street, 119121 Moscow, Russia; olbuneeva@gmail.com

**Keywords:** ubiquitin–proteasome system, atypical ubiquitination, deubiquitinase inhibitors, Parkinson’s disease

## Abstract

Ubiquitination (the covalent attachment of ubiquitin molecules to target proteins) is one of the main post-translational modifications of proteins. Historically, the type of polyubiquitination, which involves K48 lysine residues of the monomeric ubiquitin, was the first studied type of ubiquitination. It usually targets proteins for their subsequent proteasomal degradation. All the other types of ubiquitination, including monoubiquitination; multi-monoubiquitination; and polyubiquitination involving lysine residues K6, K11, K27, K29, K33, and K63 and N-terminal methionine, were defined as atypical ubiquitination (AU). Good evidence now exists that AUs, participating in the regulation of various cellular processes, are crucial for the development of Parkinson’s disease (PD). These AUs target various proteins involved in PD pathogenesis. The K6-, K27-, K29-, and K33-linked polyubiquitination of alpha-synuclein, the main component of Lewy bodies, and DJ-1 (another PD-associated protein) is involved in the formation of insoluble aggregates. Multifunctional protein kinase LRRK2 essential for PD is subjected to K63- and K27-linked ubiquitination. Mitophagy mediated by the ubiquitin ligase parkin is accompanied by K63-linked autoubiquitination of parkin itself and monoubiquitination and polyubiquitination of mitochondrial proteins with the formation of both classical K48-linked ubiquitin chains and atypical K6-, K11-, K27-, and K63-linked polyubiquitin chains. The ubiquitin-specific proteases USP30, USP33, USP8, and USP15, removing predominantly K6-, K11-, and K63-linked ubiquitin conjugates, antagonize parkin-mediated mitophagy.

## 1. Introduction

Ubiquitination is a type of common post-translational modification of cellular proteins. Ubiquitin is a protein of 76 amino acids with a molecular mass of about 8.5 kDa. Its molecules are covalently attached to the target proteins by the process of formation of a peptide bond between the carboxyl group of C-terminal residue of ubiquitin (G76) and the side chain amino group of a lysine residue of the protein substrate [1]. Different types of ubiquitination have been recognized. These include monoubiquitination (the attachment of one ubiquitin molecule to a target protein); multiple monoubiquitination, or multi-monoubiquitination (the attachment of several ubiquitin molecules); and polyubiquitination (the attachment of ubiquitin chains consisting of several ubiquitin monomers forming linear or branched polyubiquitin chains). Ubiquitin molecules may form polyubiquitin chains when they are linked by one of seven lysine residues (K6, K11, K27, K29, K33, K48, and K63) or by the N-terminal methionine residue (Met1) [2,3,4]. Protein ubiquitination is a versatile process: different types of ubiquitin chains in a compact or an open conformation (depending on the type of ubiquitin linkage) may be formed [5,6,7]. Polyubiquitin chains of the compact conformation are formed via K6, K11, and K48, while the formation of the chains of the open conformation involves K63 and M1. In the former case, a distal (C-terminal) ubiquitin molecule and a proximal ubiquitin molecule (linked via the lysine side chain amino group) require intramolecular contact sites. In the case of the open conformation, the ubiquitin molecules contact only at the linkage site. Other conformations of ubiquitin chains can also be formed, especially due to the interaction of hydrophobic isoleucine residues I36 and I44 [2,8]. 

**Figure 1:**
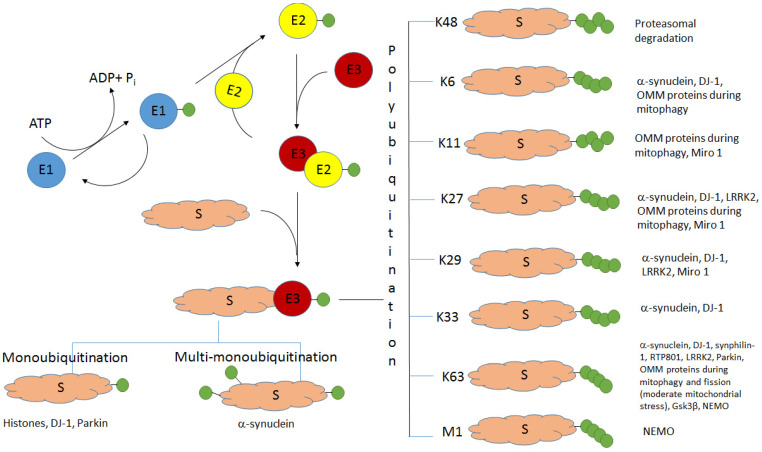
The scheme of ubiquitination and the role of different types of ubiquitination in PD. S—protein substrate, E1—ubiquitin-activating enzyme, E2—ubiquitin-conjugating enzyme, E3—ubiquitin ligase. Ubiquitin molecules are shown as green circles. Adapted from [35].

**Figure 2:**
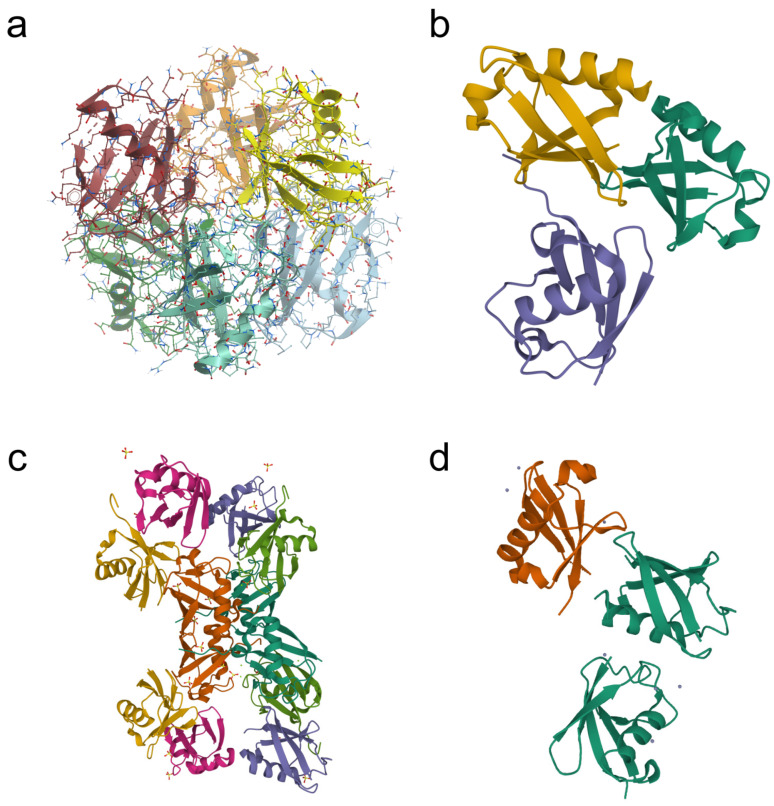
Examples of crystal structures of homotypic (**a**,**d**) and heterotypic (**b**,**c**) tri-ubiquitin chains available in the Protein Data Bank (PDB). (**a**) K27-linked tri-ubiquitin (PDB DOI: 10.2210/Pdb5jby/pdb; deposited: 14 April 2016; released: 20 July 2016; deposition authors: Pan, M., Gao, S., Zheng, Y.); (**b**) branched K11/K48-linked tri-ubiquitin (PDB DOI: 10.2210/PDB6OQ1/PDB; deposited: 25 April 2019; released: 23 October 2019; deposition authors: Boughton, A.J., Fushman, D.); (**c**) unbranched mixed tri-ubiquitin chain containing K48 and K63 linkages (PDB DOI: 10.2210/pdb5o44/pdb; deposited: 26 May 2017; released: 8 November 2017; deposition authors: Padala, P., Isupov, M.N., Wiener, R.); (**d**) K6-linked tri-ubiquitin (PDB DOI: 10.2210/pdb3zlz/pdb; deposited: 4 February 2013; released: 10 April 2013; deposition authors: Hospenthal, M.K., Freund, S.M.V., Komander, D.).

The K48-linked polyubiquitination usually targets proteins for their subsequent degradation in proteasomes (Figure 1). Historically, it was the first type of ubiquitination studied [1]. All the other types of ubiquitination were called atypical ubiquitination (AU). Ikeda and Dikic proposed a classification of atypical ubiquitin chains including all variations of multimeric ubiquitin structure, except classical K48 polyubiquitination [9]. The polyubiquitin chains formed via the same lysine residues (K6, K11, K27, K29, K33, or K63) are known as homotypic chains. The conjugation of ubiquitin monomers via different lysine residues forms the heterotypic ubiquitin chains. They can be linear or branched with specific branch points (“forks”) (Figure 2). The linear ubiquitin chains can also be generated through the alpha-amino group of the N-terminal methionine residue [10,11].

Ubiquitination requires three types of enzymes: ubiquitin-activating enzyme E1, ubiquitin-conjugating enzyme E2, and ubiquitin-protein ligase E3. The diversity of the polyubiquitin chains explains the recognition of corresponding structures by target proteins containing specific ubiquitin-binding domains [12], as well as the diversity of ubiquitin ligases (E3) and deubiquitinases cleaving the bonds between ubiquitin molecules or between the ubiquitin molecule and the target protein [13,14,15]. It appears that various combinations, including E2 and E3 enzymes and specific chaperones, are needed for linkage-specific ubiquitination [16]. 

**Figure 3:**
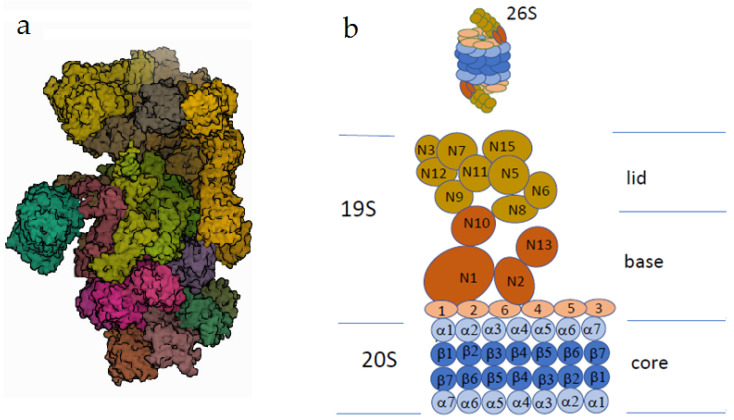
The human 26S proteasome. (**a**) Electron microscopy (PDB DOI: 10.2210/pdb5LN3/pdbEM Map EMD-4089: EMDB EMDataResource; deposited: 3 August 2016; released: 22 March 2017; deposition authors: Schweitzer, A., Beck, F., Sakata, E., Unverdorben, P. (2017) *Mol Cell Proteomics* 16: 840–854). (**b**) The scheme of the proteasome structure. One or two regulatory particles (the sedimentation coefficient 19S) may be attached to the core particle (the sedimentation coefficient 20S). The core particle consists of four rings, each of which contains seven protein subunits. The outer rings consist of alpha subunits promoting substrate insertion into the inner cavity, formed by beta subunits possessing proteolytic activity. The regulatory particle of the proteasome consists of two complexes: the lid and the base. The Rpn subunits without ATPase activity (regulatory particle non-ATPase), marked as N, form the lid. The base consists of two types of subunits. Rpt subunits of the AAA superfamily (regulatory particle triple-A ATPase) are marked as small orange ovals (Rpt1–Rpt6). Rpn subunits (marked as N) do not possess ATPase activity. The subunits N1, N10, and N13 are responsible for the recognition of the ubiquitinated substrates.

Although all types of ubiquitination, except K48, are still defined as AUs, there is accumulating evidence that AUs participate in the regulation of various cellular processes, including DNA reparation, signal transduction, and immune response [17,18,19]. Moreover, not only K48 but also some types of AU can target proteins for their proteasomal degradation. In vitro studies have shown that purified 26S proteasomes can bind and degrade K48- and K63-ubiquitinated substrates [20]. The kinetic studies of the binding of protein substrates by the proteasome revealed that three subunits of the 19S regulatory proteasome particle (known as the ubiquitin receptors) recognized protein substrates polyubiquitinated in a versatile manner [21]. Proteins with K48-linked ubiquitin chains were recognized almost exclusively by the Rpn10 subunit (Figure 3), whereas proteins targeted for degradation by atypical ubiquitin chains (K63 and certain other types) were degraded through binding to Rpn13 and Rpn1 subunits [21].

The mammalian proteasome can also bind heterotypic polyubiquitinated chains containing K11 and K48 linkages (but not homotypic K11 chains) and thus stimulate the proteasomal degradation of certain proteins [22]. However, in cells, soluble factors may selectively bind to K63 chains and inhibit/prevent their association with proteasomes [20]. Moreover, some nonubiquitinated proteins shared high affinity to regulatory protein subunits with ubiquitinated proteins [23]. Recent proteomic profiling revealed many proteins associated with the rabbit brain and liver 26S and 20S proteasomes [24,25]. The high abundance of these proteins, exceeding the abundance of intrinsic proteasomal (regulatory and core particle) proteins, suggests that the identified nonproteasomal proteins obviously represent the proteasomal subinteractome rather than contaminations [24,25]. This obviously explains altered routes of AU proteins in the cell and their destination to the cell compartments other than proteasomes. 

Convincing evidence exists that participation of AU in various cellular processes has a significant impact on the target proteins, implicated in the development of different serious diseases, including immune imbalance, cancer, and neurological problems [26,27,28]. Idiopathic or hereditary Parkinson’s disease (PD) [29] is one of the most frequent neurodegenerative disorders. It is characterized by rigidity, bradykinesia, tremor, balance problems, loss of automatic movements, and speech changes. Although most cases of PD are sporadic, gene mutations play an important role in the development of this disease. More than 20 gene mutations are associated with PD [30,31], and the list of these genes has not been completed yet [32]. Among these genes, classified as PARK-designated genes [30,31], the genes encoding proteins targeted to ubiquitination or UPS components involved in ubiquitination are especially important. These include the following: (i) the gene encoding the main components of Lewy bodies (intracellular aggregates, diagnostic markers of PD), alpha-synuclein (encoded by *SNCA*, also known as *Park1*); (ii) protein DJ-1 (encoded by *DJ-1*, also known as *Park7*); (iii) the gene encoding leucine-rich repeat kinase 2 (cytoplasmic protein known as dardarin) (*LRRK2*, *Park8*); (iv) the gene of deubiquitinase typical of brain neurons, ubiquitin C-terminal hydrolase L1 (*UCH-L1, Park5*); (v) the genes encoding E3 ubiquitin ligase parkin (*PRKN, Park2*) and phosphatase and tensin homolog (PTEN)-induced kinase 1 (*PINK1*, *Park6*) [30,31,33,34]. Mutations of most of these proteins, affecting the processes of AU, influence pathogenic mechanisms of PD (Table 1). 

Different types of AU and their role in health and disease were analyzed in our previous review [35]. Since that time, significant progress has been achieved in this field through the development of new methods of investigation [19,28,36]. In this review, we have further summarized existing information about various types of AU of proteins with an emphasis on their importance for PD.

## 2. Alpha-Synuclein and Histones: Monoubiquitination and Multi-Monoubiquitination

A typical feature of many neurodegenerative diseases is the accumulation of protein aggregates, and PD is not an exception [37]. The main component of Lewy bodies, alpha-synuclein [38,39], was shown to be subjected to monoubiquitination before its proteasomal degradation [40]. Although K48-linked polyubiquitination has been initially considered as a canonical targeting of proteins for their consequent proteasomal degradation, it becomes clear that about 40% of mammalian proteins, cleaved by proteasomes, are monoubiquitinated [41,42]. Alpha-synuclein has been shown to be monoubiquitinated by the E3 ubiquitin ligase SIAH (seven in absentia homolog) both in vivo and in vitro [43,44,45]. The ubiquitination at certain lysine residues induces structural changes in alpha-synuclein aggregates in vitro [46]. Synphilin-1A, an isoform of synphilin-1 (another component of Lewy bodies), inhibits alpha-synuclein monoubiquitination by SIAH and the formation of alpha-synuclein inclusions [47]. Rott et al. found that the deubiquitinase USP9X (downregulated in the PD substantia nigra) specifically deubiquitinates alpha-synuclein in vivo and in vitro [40]. Moreover, site-specific monoubiquitination provides different levels of alpha-synuclein degradation [48]. In contrast to monoubiquitinated alpha-synuclein, which undergoes proteasomal degradation, deubiquitinated alpha-synuclein is eliminated by autophagy [40]. Thus, USP9X regulates the degradation of alpha-synuclein [40,49]. Although it remains unclear whether protein aggregates are the cause of neuronal toxicity or the adaptation of neurons to the accumulation of toxic proteins [50], therapy modulating activity of the investigated enzymes may be a successful strategy to treat alpha-synucleinopathies. 

In the context of (pathological) protein aggregate formation, it should be noted that alpha-synuclein may form complexes with histones, protein components of chromatin, which play a key role in many cellular events [51]. Jiang et al. demonstrated the formation of such complexes and identified histones as proaggregant agents for alpha-synuclein aggregation in postmortem brains with Lewy body pathology [52,53]. Jos et al. reported that PD-specific alpha-synuclein bound histones H2A, H2B, H3, and H4 with higher affinity than wild-type alpha-synuclein [54]. 

Moreover, it has long been known that histones may be also monoubiquitinated [55,56], and histone monoubiquitination is essential for many biological pathways: transcriptional regulation [57,58,59,60], differentiation [27], cell cycle regulation [61], and DNA damage response [62,63]. Monoubiquitination of histone H2B is involved in almost all these processes [64,65]. The role of dysregulation of histone monoubiquitination in cancer progression is well known [66], but its role in neurodegeneration is also important. Permanent oxidative stress causes nuclear and mitochondrial DNA damage; PD and other neurodegenerative diseases are characterized by defective DNA repair [67,68]. Although various post-translational modifications of histones (e.g., acetylation, demethylation, phosphorylation) are intensively studied in the context of neurodegeneration [69,70], researchers still pay less attention to histone ubiquitination in these pathologies.

Monoubiquitination of histone H2A, one of the main components of chromatin and a major histone modification in mammalian cells [71,72], is crucial for neurodevelopmental disorders [59]. Histones are involved in the formation of inclusions, typical of neurodegenerative diseases, such as PD, Huntington’s disease, and Alzheimer’s disease [62,73]. Ubiquitin conjugates accumulate on the inclusion bodies, and this correlates with the depletion of ubiquitin from the nucleus, deubiquitination of histones H2A and H2B, and subsequent DNA damage [62]. Histones, typical nuclear proteins, were also discovered in mitochondrial membranes [74,75]. The role of mitochondrial dysfunction and oxidative stress in the dopaminergic neuron loss in PD is well known [76,77]. Mitochondrial toxins such as MPTP (1-methyl-4-phenyl-1,2,3,6-tetrahydropyridine) and rotenone, as well as the pesticide paraquat, are used in classical experimental models of PD [52]. Navarro-Yepes et al. demonstrated that MPTP and paraquat decreased protein ubiquitination in dopaminergic cells [78]. These results are consistent with our data on the effect of MPTP on the profile of ubiquitinated proteins of mouse brain mitochondria [79]. In addition to a decrease in the total number of ubiquitinated proteins in the brain mitochondrial fraction of MPTP-treated mice, we did not find ubiquitinated histones in this fraction. In contrast to MPTP-treated mice, the brain mitochondrial fraction of control mice contained ubiquitinated histone H2A. Both these fractions contained a histone-binding protein, histone-lysine N-methyltransferase SETD7. This protein can specifically monomethylate histone H3 and mediate the transcriptional activation of several genes [80,81]. SETD7 was ubiquitinated only in the control fraction [79]. 

We have detected histone H2A and several other histones as the components of the subproteome of the mouse brain mitochondrial Rpn10 binding proteins [23]. The brain mitochondrial fraction of control mice contained significantly fewer histones than that of MPTP-treated mice [23]. As Rpn10 is one of the ubiquitin-binding subunits of the regulatory particle of proteasome, these results are consistent with the results reported in [79].

## 3. Atypical Ubiquitination of the Components of Lewy Bodies: Alpha-Synuclein, DJ-1, and Synphilin-1

### 3.1. E3 Ubiquitin Ligase TRAF6: K6, K27, and K29 Ubiquitination of Alpha-Synuclein

Besides monoubiquitination, atypical polyubiquitination is characteristic of alpha-synuclein. It was shown that both mutant (PD-associated) and wild-type alpha-synucleins specifically interacted with the E3 ubiquitin ligase TRAF6 (tumor necrosis factor-receptor associated factor 6) and were subjected to AU via K6, K27, and K29 chains [82]. 

### 3.2. Concerted Action of the E3 Ubiquitin Ligase Parkin with the E2 Enzyme UbcH13/Uev1a: K63-Linked Ubiquitination of Alpha-Synuclein and Synphilin-1 Promotes Lewy Body Formation

The heterodimer E2 complex UbcH13/Uev1a is known to mediate K63-linked polymerization of ubiquitin. Doss-Pepe et al. have shown that parkin can function in cooperation with UbcH13/Uev1a to assemble ubiquitin-K63 chain, and alpha-synuclein can stimulate the assembly of these chains [83]. The parkin-mediated K63-linked ubiquitination of alpha-synuclein and another component of Lewy bodies, synphilin-1, contributed to the formation of synuclein/synphilin-1 inclusions [84]. Thus, K63-linked ubiquitination of alpha-synuclein and synphilin-1 could be considered as an important prerequisite for the formation of Lewy bodies [85]. 

### 3.3. HECT E3 Ligase NEDD4: K63-Linked Ubiquitination of Alpha-Synuclein

The ubiquitin ligase Nedd4 catalyzes K63-linked ubiquitination of alpha-synuclein. In vitro experiments with full-length alpha-synuclein and its recombinant fragments, lacking either the N-terminal or C-terminal residues, have shown the importance of the C-terminal residues (120–140) containing several prolines. The Nedd4 substrate interacting domain, exhibiting affinity to proline-rich motifs, bound the C-terminal region of alpha-synuclein and attached K63-linked ubiquitin chains [86]. Interestingly, the other three purified recombinant E3 ligases, implicated in alpha-synuclein degradation (CHIP, SIAH2, and parkin), were catalytically active but ineffective in alpha-synuclein ubiquitination in this in vitro system, containing either UbcH5 or UbcH7 as the E2 component [86]. In cells (over)expressing Nedd4, alpha-synuclein content decreased. The treatment of SH-SY5Y cells with selective inhibitors of proteasomes, lysosomes, and autophagy caused a 2-fold increase in alpha-synuclein content, thus suggesting several routes of alpha-synuclein degradation. Mapping the subcellular distribution of alpha-synuclein, overexpressed in neurons, revealed its association with the protein degradation pathway, including multivesicular bodies in the axons and the lysosomes within neuronal cell bodies [87]. Later, the endosomal pathway of alpha-synuclein sequestration, involving Lys-63-linked ubiquitination by the E3 ubiquitin ligase Nedd4-1, was demonstrated not only for de novo synthesized alpha-synuclein but also for internalized alpha-synuclein [88]. 

Using K63-specific alpha-synuclein antibodies, K63-linked ubiquitin conjugates were detected in alpha-synuclein-positive inclusions in postmortem brains of PD patients [89]. These inclusions were reduced in dopaminergic neurons of the substantia nigra. In contrast to K63-linked ubiquitin conjugates, the use of linkage-specific ubiquitin antibodies against Lys48 revealed only occasional staining of inclusions in the examined brain regions [89]. 

RTP801, a proapoptotic PD-related protein involved in neuronal death induction in cellular and animal models of PD, is another substrate for NEDD4-dependent K63-linked ubiquitination [90]. In a cell-free system and in cellular cultures (PC12), NEDD4 performed K63-linked ubiquitination of RTP801, thus targeting it for lysosomal degradation. The levels of NEDD4 and RTP801 changed in opposite directions in the NGF-differentiated PC12 cells treated with the neurotoxin 6-OHDA (used to model PD). The NEDD4 level decreased, while the RTP801 level increased [90]. Overexpression of wild-type NEDD4 in the NGF-differentiated PC12 cells protected cells against 6-OHDA toxicity, while the inactive mutant of NEDD4 was basically ineffective. 

Immunostaining of postmortem substantia nigra sections from PD patients and non-PD individuals (as controls) revealed loss of NEDD4 expression in remaining neurons of PD patients (but not in age-matched controls). This is consistent with the previous reports on the increase in the RTP801 level in nigral neurons [91,92]. However, in the study by Romani-Aumedes et al., RTP801 elevation was associated with the loss of parkin functioning [92].

### 3.4. DJ-1: Monoubiquitination and K63-Linked Polyubiquitination. DJ-1 and Alpha-Synuclein: K6-, K27-, and K29-Linked Polyubiquitination

DJ-1, also known as Parkinson disease protein 7, is a multifunctional protein ubiquitously expressed in cells and tissues and exhibiting both catalytic (as protein deglycase, EC 3.5.1.124) and numerous noncatalytic activities [93,94,95]. 

In cells, DJ-1 normally exists as a dimer, while its mutant forms containing amino acid substitutions, typical of PD, are characterized by impaired dimerization ability, stability, and folding [96]. The amino acid substitution L166P has the most pronounced devastating effect on the DJ-1 structure, folding, and homodimerization [97]. Being detected in the cytoplasm, DJ-1 may be translocated into mitochondria and the nucleus. In mitochondria, DJ-1 interacts with NADH dehydrogenase and ATP synthase subunits and plays an important role in the functional integrity of the inner mitochondrial membrane. In the nucleus, DJ-1 functions as a histone deglycase [98] and interacts with (and sequesters) the Daxx protein, thus preventing cell death [99]. DJ-1 has been also recognized as a coactivator of various signaling pathways of the androgen receptor [100,101], Nrf2 [102], and p53 protein [103,104]. It is also essential for transcriptional activation of the tyrosine hydroxylase gene, encoding the key enzyme of dopamine synthesis [105,106]. The biological activity of DJ-1 may be regulated by small ubiquitin-like modifiers (SUMOs) [107,108]. In cells, wild-type DJ-1 undergoes sumoylation at the lysine residue (K130); in the case of the L166P mutation associated with the development of PD, sumoylation occurred at other lysine residues with the formation of misfolded insoluble forms of the protein [107]. 

In neuroblastoma cells and in human brain lysates, DJ-1 forms a complex with parkin and PINK1 [109]. This complex has ubiquitin ligase activity, which ubiquitinates parkin substrates (for example, synphilin-1) and parkin itself [109]. However, parkin does not ubiquitinate wild-type DJ-1 [110]. Genetic depletion of DJ-1 or PINK1 decreased the ubiquitination level of parkin and its substrates and caused the accumulation of aberrant proteins. 

DJ-1 may also interact with another E3 ubiquitin ligase [111] known as a tumor suppressor, VHL (von Hippel–Lindau) protein [112,113]. This interaction impairs VHL interaction with the alpha subunit of the heterodimeric hypoxia-inducible transcription factor 1 (HIF-1alpha) and prevents the ubiquitination of HIF-1alpha. DJ-1 deficiency leads to a decrease in the HIF-1alpha level during hypoxia and oxidative stress. Lymphoblasts of patients with PD, associated with DJ-1 mutations, are characterized by less stable HIF-1alpha compared to that in healthy people. This suggests that DJ-1 protects neurons from death by inhibiting the VHL ubiquitin ligase activity [111]. 

DJ-1 can serve as a regulator of the 20S proteasome. DJ-1 binding to the proteasome inhibits its activity, and this prevents the degradation of partially unfolded proteins [114]. This protects some important protein substrates of the 20S proteasome (for example, alpha-synuclein and p53) against proteasomal degradation, thus maintaining their steady-state cellular level. Under oxidative stress, DJ-1 exhibits the opposite effect and activates the 20S proteasome via the Nrf2 (nuclear factor erythroid 2-related factor 2)-dependent signaling pathway, facilitating rapid removal of the damaged proteins from the cell [114]. In the context of the functioning of the ubiquitin–proteasome system, DJ-1 is obviously involved in a network interaction of ubiquitinated proteins [115]. A recent analysis of the mouse brain mitochondrial ubiquitylome [115] has shown that the network interaction of ubiquitinated proteins is formed by three functional horizontal layers linked by three proteins, including DJ-1 [115]. 

In contrast to the wild-type protein, the mutant DJ-1, containing the amino acid substitution L166P found in PD patients, may be ubiquitinated by several E3 ubiquitin ligases. Parkin selectively recognizes and ubiquitinates L166P mutant DJ-1, but not wild-type DJ-1 [110]. Using several ubiquitin mutants (containing arginine substitutions of all its lysine residues, except just one lysine at corresponding position 29, 48, or 63), the authors demonstrated K63-linked polyubiquitination [110]. In vitro ubiquitination reactions, performed using the ubiquitin mutants, revealed the accumulation of the monoubiquitinated L166P mutant DJ-1. Since parkin overexpression has no impact on the steady-state level of both L166P mutant and wild-type DJ-1, it appears that K63-linked polyubiquitination targets L166P mutant DJ-1 for the pathways other than proteasomal degradation [110]. 

The E3 ubiquitin ligase TRAF6 (TNF receptor-associated factor 6) ubiquitinates misfolded mutant DJ-1 and mutant alpha-synuclein, the main component of Lewy bodies [82,116]. Interestingly, TRAF6 performs atypical polyubiquitination of DJ-1 and alpha-synuclein due to the lysine residues K6, K27, and K29 of the ubiquitin molecule. This promotes the accumulation of insoluble aggregates of polyubiquitinated DJ-1 and alpha-synuclein in the cytoplasm. In the postmortem brains of PD patients, TRAF6 was colocalized with DJ-1 and alpha-synuclein [82]. 

The level of polyubiquitinated mutant DJ-1 (L166P) but not wild-type DJ-1 in cells was sensitive to the proteasome inhibitor MG132 [117]. In the MG132-treated cells, the steady-state level of the L166P mutant increased, thus suggesting mutant DJ-1 degradation by the ubiquitin–proteasome system [117]. 

Parkin-mediated (in cooperation with the heterodimeric E2 UbcH13/Uev1a) K63-linked polyubiquitination of misfolded DJ-1 serves as a signal for binding of histone deacetylase (HDAC6) [110]. HDAC6 is involved in aggresome formation: it binds polyubiquitinated (misfolded) proteins and the dynein motors, thus recruiting the misfolded protein cargo to dynein motors transporting to aggresomes [118]. HDAC6-deficient cells cannot clear misfolded protein aggregates from the cytoplasm and are hypersensitive to misfolded protein accumulation [118]. In this context, HDAC6 did not bind nonubiquitinated L166P mutant DJ-1, but it effectively interacted with K63-polyubiquitinated L166P mutant DJ-1 in cells treated with the proteasome inhibitor MG132 [110]. 

The other DJ-1 mutant, with amino acid substitution T154A preventing phosphorylation of this protein by protein kinase A at the T154 residue, demonstrated much higher sensitivity to proteasomal degradation than wild-type DJ-1 [119]. 

## 4. LRRK2: K63- and K27-Linked Ubiquitination

Leucine-rich repeat kinase 2 (LRRK2), a member of the leucine-rich kinase family, is a multifunctional protein kinase containing a GTPase domain. Mutations in the corresponding gene (*LRRK2*, also known as *PARK8*) are linked to the most common familial forms and some sporadic forms of PD, and small-molecule inhibitors of LRRK2 attract much interest in the context of PD therapy [120,121,122]. 

LRRK2 is a substrate for the carboxyl terminus of HSP70-interacting protein (CHIP) known as CHIP E3 ligase, which regulates the steady-state level of LRRK2 via UPS degradation [123]. The LRRK2 level was significantly (4-fold) higher in the soluble fraction of the brains of CHIP knockout mice than in control wild-type mice [123]. The cells treated with a CHIP siRNA exhibited a higher level of LRRK2 (by 20–25%), while the cells exposed to the wild-type CHIP were characterized by a detectable decrease in the LRRK2 level (20–25%) [123]. Although the type of LRRK2 ubiquitination by CHIP has not been investigated, studies with other protein targets indicate that CHIP enhances K63-linked ubiquitination [124]. CHIP-dependent ubiquitination of LRRK2 involves interactions with several heat shock proteins [123,124], particularly HSP90, which is one of the three key players (together with DJ-1 and SOD2) forming a single network of brain ubiquitinated proteins [115]. The 20S proteasome inhibitor, clasto-lactacystin beta-lactone, increased the level of ubiquitinated LRRK2 in HEK293 cells, while CHIP overexpression in SH-SY5Y cells attenuated the toxicity of G2019S and R1441C LRRK2 mutants [123]. 

The other E3 ubiquitin ligase WSB1 (WD repeat and SOCS box-containing protein 1) could ubiquitinate LRRK2 through K27- and K29-linkage chains [125]. This was accompanied by LRRK2 aggregation and neuronal protection in primary neurons and LRRK2-G2019S transgenic Drosophila flies. Analysis of postmortem brains of PD patients revealed colocalization of WSB1 and LRRK2 in the Lewy bodies. Moreover, almost all Lewy bodies (97%), identified by alpha-synuclein staining, had WSB1 reactivity, and about 25% of Lewy bodies contained LRRK2 [125]. This was specific for PD but not for Alzheimer’s disease, as WSB1 reactivity was not detected in plaques [125]. 

Functional inhibition of the LRRK2 GTPase domain and kinase activity by GTP-binding inhibitors (compounds 68- or Fx2149) increased G2019S-LRRK2 polyubiquitination at K27- and K63-linkages in HEK293T cells cotransfected with LRRK2 constructs and various HA-tagged ubiquitin constructs [126]. Both compounds increased G2019S-LRRK2-linked ubiquitinated aggregates, thus underlying a critical role of the GTP-binding for LRRK2-linked ubiquitination and formation of aggregates [126]. Treatment of C57BL/6J mice with Fx2149 (10 mg/kg intraperitoneally twice daily for three days) significantly increased endogenous ubiquitination of brain LRRK2 [126]. 

Table 2 summarizes current knowledge on AU of PD-related proteins. 

## 5. Concerted Mechanisms of Different Types of Ubiquitination and Deubiquitination in Mitophagy (Monoubiquitination and K6-, K11-, K27-, and K63-Linked Polyubiquitination)

Studies of postmortem brain samples of PD patients and experimental PD models provided convincing evidence for the critical role of mitochondrial dysfunction and oxidative stress in the neuronal loss in PD [145,146,147,148,149,150]. Elimination of damaged mitochondria (mitophagy) is of vital importance for both neighboring mitochondria and the whole cell [151,152]. The proteins related to PD, PINK1 (serine/threonine protein kinase) and parkin (E3 ubiquitin ligase), are the major players in mitophagy regulation. The mechanism of mitophagy has been considered in several recent reviews [151,153,154]. The role of post-translational modifications of proteins (particularly ubiquitination) in this cascade cannot be overestimated [132]. Various mitochondrial proteins are subjected to monoubiquitination and K6-, K11-, K27-, and K63-linked polyubiquitination by the E3 ligase parkin and deubiquitination by the mitochondrial deubiquitinase USP30.

Under normal conditions (with the high value of mitochondrial membrane potential), PINK1 is translocated to the mitochondrial inner membrane via TOM (translocase of outer membrane) and TIM (translocase of inner membrane) complexes [155] and degraded after a series of proteolytic cleavages. This process represses mitophagy. Mitochondrial depolarization induced by mitochondrial poisons (MPTP, the insecticide rotenone, or the herbicide paraquat) blocks PINK1 import and triggers its accumulation on the outer mitochondrial membrane (OMM) [156]. Being accumulated on the OMM, PINK phosphorylates both parkin (S65 of the N-terminal ubiquitin-like domain) [157] and ubiquitin moieties of ubiquitin conjugates attached to certain ubiquitin-modified OMM proteins [158,159]. This phosphorylation activates parkin and stimulates its recruitment from the cytosol to mitochondria via direct binding of phosphoubiquitin conjugates on the mitochondrial surface. Active parkin promotes polyubiquitination of various mitochondrial substrates; this causes phosphorylation of their ubiquitin conjugates by PINK1, further parkin recruitment, activation, and ubiquitination of protein substrates by a feed-forward mechanism [132,151]. 

The combination of techniques (cells expressing GFP-parkin, use of uncouplers causing mitochondrial depolarization, antibodies specific for polyubiquitin species, Western blot analysis, and mass spectrometry) has led to the identification of the protein substrates ubiquitinated by parkin and the type of ubiquitination [127,128,129,130,131,132,133]. In the context of an extraordinarily large number of parkin substrates, recognized in proteomic studies, parkin is considered as a promiscuous E3 ligase; its flexible specificity is based more on the presence of phosphoubiquitin on the surface of substrates rather than on the identity of the substrates per se [132]. Moreover, parkin is even considered as “the first phospho-ubiquitin-dependent E3” [132]. 

Only a part of the identified parkin substrates turned out to be ubiquitinated with classical K48-linked ubiquitin chains (destined for proteasomal targeting), whereas most of them were targeted with atypical K6, K11, and K63 ubiquitin chains [129,133,134,135,136,160,161,162,163,164,165]. These proteins are localized in the OMM and play a significant role in the regulation of membrane transport and oxidative phosphorylation. They are responsible for the crosstalk between mitochondria and the rest of the cell, apoptosis, mitochondrial membranes organization, cell differentiation, and fusion and fission of mitochondria (see Table 3). Parkin self-ubiquitination also proceeded with the formation of not only K48, but also K6, K11, and K63 ubiquitin chains [130]. 

Some of the above-mentioned mitochondrial membrane proteins are subjected to less common types of ubiquitination. For example, mitochondrial GTPase Miro1 undergoes not only K11-, but also K27- and K29-linked polyubiquitination [137]. While ubiquitinating VDAC1, one of the key proteins regulating mitochondrion function and the most abundant protein of the outer mitochondrial membrane [166], parkin formed not only K6-, K11-, and K63- [129] but also K27-linked ubiquitin chains [133]. 

Recently, using a Drosophila PD model, Ham et al. have shown that VDAC1 was monoubiquitinated by parkin [135]. The authors emphasize the critical role of VDAC1 monoubiquitination by parkin in a PINK1-dependent manner in the regulation of mitophagy and apoptosis and, therefore, in the pathogenesis of PD. Monoubiquitination of VDAC1 decreases calcium entry into mitochondria, thus preventing apoptosis and promoting mitophagy; the authors look forward to finding proper control of VDAC1 monoubiquitination to prevent and treat PD [135].

## 6. SCFFbxo7/PARK15 Ubiquitin Ligase: K63-Linked Ubiquitination 

Fbxo7 (F-box only protein 7) is an F-box protein associated with PD. Mutations in its gene *FBXO7* (also known as *PARK15*) (Table 1) cause autosomal recessive, early-onset PD [167,168]. The interaction of Fbxo7 with two PD-related proteins (PINK1 and parkin) is essential for mitophagy [169]. In transfected HEK293T and SHSY-5Y KD cells, Fbxo7 catalyzed predominantly K63-linked ubiquitination [139]. The studies with the Fbxo7 substrate, Gsk3β, have shown that ubiquitination of this enzyme negatively regulated its activity but not its localization. The level of Gsk3β in the cell remained basically unchanged even under conditions of reduced Fbxo7 expression [139], thus suggesting an important regulatory role, rather than elimination of the polyubiquitin-labeled protein.

## 7. UBE2N, UBE2L3, and UBE2D2/3 as Atypical Ubiquitin Chains Forming E2 Ubiquitin-Conjugating Enzymes

The parkin-mediated mitophagy requires particular E2 (ubiquitin-conjugating) enzymes. The heterodimer consisting of UBE2N and UBE2V catalyzes the formation of K63-linked ubiquitin chains, while UBE2L3 forms predominantly K11-linked chains [170]. The simultaneous knockdown of three E2 enzymes (UBE2N, UBE2L3, and UBE2D2/3) significantly impaired the ubiquitination of mitochondrial proteins and mitophagy [171]. This suggests that UBE2N, UBE2L3, and UBE2D2/3 are the key regulators of parkin-depended mitophagy and they function in a cooperative manner [171].

## 8. Deubiquitinases (USP8, USP15, USP30, USP33, USP35, UCH-L1) Removing Atypical Ubiquitin Conjugates from PD-Related Proteins

Deubiquitinases (DUBs) are known to be another factor of mitophagy regulation [172,173,174,175]. There are more than 90 different human DUBs [176]. These include several enzymes cleaving peptide bonds of atypical ubiquitin chains and thus regulating mitophagy [132,151,177]. Some of these enzymes deubiquitinate parkin itself, while the others act on the ubiquitinated protein substrates of parkin. USP8 regulates parkin autoubiquitination during mitophagy [130]. The USP8 silencing impaired mitophagy: the U2OS-GFP-parkin cells transfected with USP8 siRNA did not demonstrate a loss of mitochondrial staining of parkin substrates. USP8 deubiquitinated parkin itself by selectively removing K6-linked ubiquitin conjugates from it [130]. Interestingly, in contrast to mitophagy promotion, USP8 seems to play an opposite role in the context of autophagy: USP8 interacts with alpha-synuclein and cleaves its K63 ubiquitin conjugates, preventing alpha-synuclein degradation in lysosomes [89]. Thus, USP8 represents a rare example as a DUB that regulates mitophagy positively, whereas most of the known deubiquitinases (USP33, USP15, USP35, USP30) regulate it negatively [178,179,180,181].

Another DUB, USP33, deubiquitinated parkin mainly at Lys435, removing not only K48, but also atypical K6-, K11-, and K63-linked ubiquitin conjugates. Moreover, USP33 silencing protected human neuroblastoma cells from MPTP-induced apoptotic death [178]. USP15 was identified as a DUB counteracting parkin-mediated ubiquitination of mitochondrial proteins [179]. Overexpression of this enzyme antagonized the clustering of both K48- and K63-linked ubiquitin chains on depolarized mitochondria. Knockdown of USP15 prevented mitophagy of PD patient fibroblasts with mutations in the parkin-encoding gene and decreased parkin levels [179]. 

Ubiquitin C-terminal hydrolase L1 (UCH-L1) is genetically associated with PD [182] (Table 1). UCH-L1, downregulated in PD patients [183] and found in Lewy bodies of autopsy brains [184], is also involved in the deubiquitination of atypical ubiquitin chains. McKeon et al. studied in vivo and in vitro UCH-L1 ubiquitination using mutant forms of parkin and UCH-L1, SH-SY5Y or HeLa cells, and parkin knockout mice. They found that parkin along with the ubiquitin-conjugating E2 complex Ubc13/Uev1a (typical of building K63 ubiquitin chains) mediated K63-linked polyubiquitination of UCH-L1, thus promoting UCH-L1 degradation through the autophagy–lysosomal pathway [185]. 

Meray et al. studied UCH-L1 ubiquitination using COS7 cells and transfection technique with plasmids encoding human UCH-L1 and human ubiquitin, Western blot, and immunostaining [186]. They demonstrated reversible monoubiquitination of UCH-L1 in cells at a lysine residue near the active site. The monoubiquitination inhibited the binding of free ubiquitin or ubiquitinated targets in vitro, thus inhibiting UCH-L1 capacity to increase free ubiquitin levels in the cells. The monoubiquitination of UCH-L1 was reversed by its auto-deubiquitination, modulating the lifetime of this modification of the enzyme. In addition to the hydrolase activity, UCH-L1 possesses an opposite enzyme activity, acting as a ligase. This unusual function of UCH-L1 promotes K63-linked polyubiquitination of alpha-synuclein and, as a result, alpha-synuclein accumulation in the cell [187]. Since parkin-mediated K63-linked ubiquitination of synphilin-1 and alpha-synuclein results in the formation of Lewy body-like inclusions [188], it has been suggested that the formation of inclusions typical of PD and other conformational diseases is enhanced under the conditions promoting cellular K63-linked polyubiquitination [85]. 

## 9. M1-Linked Ubiquitination

The linear ubiquitin chain assembly complex (LUBAC), composed of heme-oxidized IRP2 ligase-1 (HOIL-1L) acting as an E3 ligase, associated with HOIP (HOIL-1L-interacting protein) and SHANK-associated RH domain interacting protein (SHARPIN), generates a novel type of Met1 (M1)-linked linear polyubiquitin chain [144]. Certain evidence exists that LUBAC is the only E3, linking linear polyubiquitin chains by peptide bonds between the C-terminal G76 of ubiquitin and the α-NH2 group of M1 of another ubiquitin [144]. 

There is evidence that LUBAC assembles M1-linked ubiquitin preferentially on pre-existing K63-linked ubiquitin and some other linkages [189,190]. LUBAC may be (perhaps indirectly) implicated in PD. At least, it was found that parkin activated LUBAC-mediated linear ubiquitination of essential modulator (NEMO) by modifying this modulator with K63-linked ubiquitin [142]. Other scenarios include LUBAC-mediated M1 linear ubiquitination of multiple components of the NF-κB signaling pathway and mitogen-activated protein kinases (NEMO, etc.) [141,143]. 

## 10. Conclusions

Many proteins undergoing atypical mono- and polyubiquitination (or implicated in such ubiquitination) and involved in different biological pathways are related to PD. A significant proportion of such proteins are the products encoded by the genes associated with the familial forms of PD (Table 1). The results of numerous studies, considered in this review, provide some hints for new therapeutic intervention of PD. For example, the elimination of damaged mitochondria could involve the regulation of decision between mitophagy and apoptosis via VDAC1 monoubiquitination or polyubiquitination by parkin [135]. 

Among the enzymes involved in the regulation of the ubiquitination/deubiquitination process, the deubiquitinase USP30 attracts much interest as a promising therapeutic target. USP30 functions as an important negative regulator of mitophagy [129,134,136,175,180,181,191,192]. By deubiquitinating K6- and K11-linked ubiquitin chains of parkin, USP30 delays mitophagy. Studying a series of compounds as USP30 inhibitors, Kluge et al. [193] found the most potent compound was MF094, *N*-(1-((5-(*N*-(*tert*-butyl)sulfamoyl)naphthalen-1-yl)amino)-1-oxo-3-phenylpropan-2-yl)cyclohexanecarboxamide, which inhibited USP30 with the IC_50_ value of 120 ± 0.26 (nM). The effect of USP30 inhibition by MF094 was accompanied by increased protein ubiquitination and accelerated mitophagy. 

Some DUB inhibitors have been found among isatin O-acyl oximes [194]. These are especially interesting because various components of the UPS (as well as some PD-related proteins) have been identified earlier as isatin-binding proteins [195,196]. Isatin is an endogenous indole exhibiting various biological and pharmacological activities [197]. By interacting with numerous intracellular isatin-binding proteins, isatin can act as a regulator of complex protein networks in normal and pathological states [93,115,197]. 

When administered in vivo, isatin attenuates manifestations of MPTP-induced parkinsonism in experimental animal models [23,79,195,196,197]. Considering its interaction with various proteins of the UPS, it would be interesting to investigate various isatin analogs for their possible activity as regulators of the UPS and AU and as potential antiparkinsonian agents. 

## Figures and Tables

**Table 1:** Some PARK-designated genes and their protein products involved in inherited forms of Parkinson’s disease (modified from [30,31] and supplemented).

Symbol	Gene	Protein Product	Relation to UPS	Type of Disease	Inheritance
*PARK1*	*SNCA*	Alpha-synuclein	Ubiquitination substrate	Classical and early-onset PD	AD *
*PARK2*	*Parkin*	*Parkin*	E3 ubiquitin ligase	Early-onset PD	AR **
*PARK5*	*UCHL1*	*Ubiquitin C-terminal hydrolase L1*	Deubiquitinase	Classical PD	AD
*PARK6*	*PINK1*	PTEN-induced kinase 1	Phosphorylates ubiquitination substrate E3 ubiquitin ligase	Early-onset PD	AR
*PARK7*	*DJ-1*	*DJ-1*	Ubiquitination substrate	Early-onset PD	AR
*PARK8*	*LRRK2*	Leucine-rich repeat kinase 2	Ubiquitination substrate	Classical PD	AD
*PARK10*	*USP24*	Ubiquitin-specific peptidase 24	Deubiquitinase	Late-onset PD	Risk factor
*PARK11*	*GIGYF2*	Grb10-interacting GYF protein-2	Could promote ligand-induced ubiquitination of IGF1R	Classical PD	AD

* AD, autosomal dominant; ** AR, autosomal recessive.

**Table 2:** Atypical ubiquitination of proteins in PD and PD-related models.

Target Protein	Type of Ubiquitination	Enzymes Studied (E2, E3, DUBs, etc.)	Biological Effect	References
Histones H2B, H2A	Monoubiquitination	Not studied	Deubiquitination of histones H2A and H2B correlated with the accumulation of ubiquitin conjugates on the inclusion bodies and DNA damage. In contrast to control, brain mitochondria of MPTP-treated mice did not contain ubiquitinated histone H2A.	[62,79]
Alpha-synuclein	Multiple monoubiquitination	E3 ligase SIAH deubiquitinase USP9X	Alpha-synuclein monoubiquitination promoted aggregate formation in vitro and in vivo. Site-specific monoubiquitination provided different levels of alpha-synuclein degradation. USP9X regulated alpha-synuclein degradation.	[43,44,45,46,48,49]
Alpha-synuclein, proapoptotic PD-related protein RTP801	K63-linked polyubiquitination	HECT E3 ligase NEDD4	Nedd4 catalyzed K63-linked ubiquitination of alpha-synuclein in cells. K63-linked ubiquitin conjugates were detected in alpha-synuclein-positive inclusions in postmortem brains of PD patients. In cells (over)expressing Nedd4, alpha-synuclein content decreased. In the cell model of PD, 6-OHDA decreased NEDD4 and increased RTP801.	[86,87,88,89,90]
Alpha-synuclein and synphilin-1	K63-linked polyubiquitination	E3 ligase parkin, E2 enzyme UbcH13/Uev1a	K63-linked ubiquitination of alpha-synuclein and synphilin-1 promoted Lewy body formation.	[83,84,85]
Alpha-synuclein, DJ-1	K6-, K27-, and K29-linked polyubiquitination	E3 TRAF6	TRAF6 interaction with mutant DJ-1 and alpha-synuclein promoted the formation of atypical ubiquitin chains and insoluble DJ-1 aggregates.	[82,116]
DJ-1	Monoubiquitination, K63-linked polyubiquitination	E3 ligase parkin, PINK1, E3 ubiquitin ligase VHL	K63-linked polyubiquitination targets L166P mutant DJ-1 for the pathways other than proteasomal degradation. Parkin overexpression had no impact on the steady-state level of both L166P mutant and wild-type DJ-1.	[110]
LRRK2	K63-, K27-, and K29-linked polyubiquitination	E3 ubiquitin ligase CHIP (K63-) E3 ubiquitin ligase WSB1 (K27-, K29-)	LRRK2 is a substrate for CHIP, which regulates the steady-state level of LRRK2 via UPS degradation. WSB1 ubiquitinates LRRK2 through K27- and K29- linkage chains followed by LRRK2 aggregation and neuronal protection in primary neurons.	[124,125,126]
E3 ligase parkin. Outer mitochondrial membrane (OMM) proteins	Monoubiquitination. K6-, K11-, K27-, and K63-linked polyubiquitination and deubiquitination	PINK1; E3 ligase parkin; UBE2N; UBE2L3; UBE2D2; DUBs USP8, USP15, USP30, USP33, USP35, UCH-L1	In response to OMM depolarization, parkin (phosphorylated by PINK1) was autoubiquitinated (K63) and ubiquitinated mitochondrial proteins with the predominance of K11, K63, and K6 chains (with subsequent mitophagy). Deubiquitination of mitochondrial proteins negatively regulated mitophagy.	[127,128,129,130,131,132,133,134,135,136]
Miro1 GTPase	Predominantly K27- and some K11- and K29-linked polyubiquitination	PINK1, parkin	Mitochondrial damage caused parkin phosphorylation by PINK1, followed by K27-linked ubiquitination of the outer membrane Miro1, and retarded proteasomal degradation of Miro1.	[137]
Mitochondrial proteins	K63-linked polyubiquitination	E3 ligase parkin, E2 Ubc13	Under moderate mitochondrial stress conditions, parkin provides mitochondrial connectivity causing mitochondrial fission by catalyzing (together with E2 Ubc13) its K63-linked ubiquitination.	[138]
Glycogen synthase kinase 3 beta (Gsk3beta)	K63-linked polyubiquitination	SCFFbxo7/PARK15 ubiquitin ligase	The ubiquitination of the enzyme Gsk3beta negatively regulated its activity but not its localization.	[139]
NEMO, components of the NF-κB signaling pathway, and MAP kinases	M1-linked ubiquitination, K63-linked polyubiquitination	E3 ligase LUBAC	Increase in LUBAC-mediated M1-linked (linear) ubiquitination of NEMO	[140,141,142,143,144]

**Table 3:** Atypical ubiquitination of mitochondrial proteins *.

Accession (Swiss-Prot)	Gene	Description (Swiss-Prot)	Function
Q9NZ45	*CISD1*	CDGSH iron sulfur domain-containing protein 1	Regulation of electron transport and oxidative phosphorylation
Q14318	*FKBP8*	Peptidyl-prolyl cis-trans isomerase FKBP8	Apoptosis regulation, host–virus interaction
Q8TB36	*GDAP1*	Ganglioside-induced differentiation-associated protein 1	Regulates the mitochondrial network by promoting mitochondrial fission
GPKOW	*MOS2*	G-patch domain and KOW motifs-containing protein	RNA-binding protein involved in pre-mRNA splicing
Q13505	*MTX1*	Metaxin-1	Transport of proteins into the mitochondrion
Q969V5	*MUL1*	Mitochondrial ubiquitin ligase activator of NFKB 11	Control of mitochondrial morphology by promoting mitochondrial fragmentation
Q9Y3E5	*PTRH2*	Peptidyl-tRNA hydrolase 2, mitochondrial	Promotes caspase-independent apoptosis by regulating AES and TLE1
Q15388	*TOMM20*	Mitochondrial import receptor subunit TOM20 homolog	Central component of the receptor complex responsible for import of protein precursors into mitochondria
O96008	*TOMM40*	Mitochondrial import receptor subunit TOM40 homolog	Channel-forming protein essential for import of protein precursors into mitochondria
O94826	*TOMM70*	Mitochondrial import receptor subunit TOM70 homolog	Recognizes and translocates mitochondrial preproteins from the cytosol into the mitochondria
Q9Y2W6	*TDRKH*	Tudor and KH domain-containing protein	Participates in the primary piRNA biogenesis pathway
Q8IWA4	*MFN1*	Mitofusin-1	Mitochondrial outer membrane GTPases that mediate mitochondrial clustering and fusion
O95140	*MFN2*	Mitofusin-2	
P21796	*VDAC1*	Voltage-dependent anion-selective channel protein 1	Isoforms of the outer membrane integral pore-forming multifunctional protein. Regulate the exchange of a variety of metabolites (including ATP and ADP), thus controlling crosstalk between mitochondria and the rest of the cell
P45880	*VDAC2*	Voltage-dependent anion-selective channel protein 2	
Q9Y277	*VDAC3*	Voltage-dependent anion-selective channel protein 3	
Q8IXI2	*RHOT1*	Mitochondrial Rho GTPase 1 (Miro1)	Mitochondrial GTPase involved in mitochondrial trafficking

* Compiled from [127,128,129,130,131,132,133,134,135,136,137].
